# Ultraism in Music

**Published:** 1879-10

**Authors:** Ben. H. Pratt


### THE BISTOURY.

Vol. XV. ELMIRA, N. Y., OCTOBER, 1879. No. 3.

### ULTRAISM IN MUSIC.

BEN. H. PRATT.

A pleasure may be carried to such an excess of refinement as to become not only a dread, but an absolute torture. The true province of art is to take the crude and the uncouth, and mould it into the complete and the beautiful. There its mission ends. But the tendency of art is to ultraism—something beyond that which is desirable.

There is a wide range of domestic joys—wide as the human race—to which we might apply the assertion just made. I will take but a single one of these at this time—one familiar to all—one which daily presents itself to all—one which art is fast converting into a disturber of our peace rather than a contributor to our happiness.

Music is the solace of mankind—has been since the days of Jubal. It was born of the joyous gush of the human soul. It is the vocalized expression of joy itself, just as weeping is that of sorrow. However crude it may have been in the beginning, it doubtless gave to him who evoked it, and to him who listened to it, a rare sense of enjoyment. Art, co-eval with sound itself, at once sprang to the aid of music, and developed the voice of primeval man into a rude species of song; the whistling of the wind among the branches of trees into stringed instruments, and the accidental discovery of tones in reeds into tuneful pipes. Art and music then went hand in hand adown the ages, until they reached the maximum of sound—evoking joy. Music became the language of pathos. Soul revealed itself to soul through its medium as it could reveal itself in no other way. By means of certain combinations of musical tones, the voice or the instrument revealed emotions which were at once caught and rekindled in the soul of the hearer. More than this, the range of musical expression was wider than speech, and deeper even than the thought of which it was the offspring. It became the only medium of emotional transmission. It became a joy, a thrill, an ecstasy. Harmony was such in truth as it is now in name only. It was real, whereas now it is technical and false. Art, having accomplished so much, and unable to resist the temptation to still further develop the gifts of its handmaid, plied the process still further, and conducted music so far beyond and above the mental comprehension as to make it unintelligible to all save the few whom the deceiver vainly strove to educate up to her own supernatural standard.

To-day, that which we call music lacks appreciation, simply because it is too artistic. Tune is out of fashion. Now and then a straggling air is let into our music, but it is strangled to death before it ever gets out again. Even airs are scarcely tolerated. Those bewitching melodies that used to set us all aquiver with delight—that we could not only discover as soon as the voice reached the ear, but couldn't help absorbing at once—that never got away from us until the sound ceased—that ran in our heads and were hummed for days after they were heard—these are now *passe*, and accounted among vulgarisms of the past. In their stead we have the screechful opera, with its squall, and growl, and shriek—the gymnastic pianism, with its thumps and bang and meaningless triddle. The singer of to-day is he or she who can utter the most unnatural sounds that the ingenuity of man and the capability of windpipe can devise and accomplish. Not content with this, our ears must be mocked with words from a foreign tongue or no tongue at all, and inanity of the sublimest sort must characterize the vocalist of the time, or the performer is doomed to dire condemnation. And people will sit and try to listen to it all. They will suffer the torments of a stiff-backed, cramped-up seat for hours, in order to be accounted patrons of the musical muse. It is the fashion to be bamboozled, and the screechier and squallier the bamboozler, the happier seems the victim. We must they say, become educated up to this crazy standard, for "with many stripes are we healed." We must not only hear and endure, but we must go off into raptures over these things. We must say "how delightful!" when we mean "how cursed!" We must suffer, and lie, and deceive our very selves, if we can. We know it is the very essence of absurdity and tomfoolery for any woman or man to strain themselves for an hour at a time to see how much a throat can stand without splitting, or a pair of lungs can endure without rupturing; and we know, too, that we are still bigger fools for encouraging them in it and praising them in it. We might pity the deluded things if we wern't more deluded than they.

To prove that this sort of thing is not singing, let one of these famous vocal contortionists, after having punished herself and her hearers with one of those things they call an *aria di tracheasplitter*, or something of that sort, sing one of the good old songs that is sweetness and beauty from beginning to end. Before the audience has had time to reflect upon the vulgarity of applauding a thing of this sort, it will, spasmodically, and regardless of all the proprieties, have fairly applauded itself into a fever of delight. The one is the result of hypercultivation in an unnatural direction, and the other is the spontaneous outburst of uncontrollable admiration. The one is forced, the other is voluntary.

I have sat in a fashionable theatre, among a fashionable audience, and heard a celebrated pianist execute the most wonderful music that could be imagined. And there were whispered "oh's," and "ah's," and "delightful's," and "perfectly splendid's," and "exquisitly lovely's" whizzing by my ears during the first half of the performance. There were murmurings of delight everywhere and the murmurs grew more and more audible, and pretty soon everybody was talking half aloud, and the topics of conversation had by degrees deserted the pianist and the playing, and had got into the realm of dress and gossip and everything else except this special performance, when suddenly the piano ceased its unintelligible uproar, and the whole house was caught doing everything but listening. It was simply a *faux pas*. The audience forgot itself. It meant to be entranced by the time the pianist was ready to leave the stool, but the stop came too quick for it, and the chagrin thereover was awful. An attempt was made to recover and applaud, but it was too artificial a thing to be done well, and the house laughed instead, at its own hypocrisy, and the farce ended in a roar of chagrin.

Again, there are very few persons who have not been present at a private evening entertainment, and among those invited to "play something," have noticed a vast difference in the styles of young lady pianists. I am free to say that there are, among the pupils of even the most hyperartistic teachers of piano music, those whom no instructor can spoil—those whose inate conception of the true in music cannot be overcome by all the wiles of the preceptor. They are few, but there are such. At such an entertainment as that referred to, those who are esteemed by their teachers as their best pupils—their prodigies—those of whom they brag—they who are always brought out to show off the efficiency of the instructor upon every occasion—these are the very girls whom all listen to with rapt attention for a bar or two, after which they are left to complete their performance amid a buzz and hum of voices that are a downright insult to the lady at the instrument, and they all know it, and the player knows it, and yet both player and auditors know too that it isn't expected that people will care for that which they can't understand, can't appreciate and would gladly have abolished if it wasn't for the name of the thing. That which was intended to entertain has become a source of discomfort, and instead of conveying pleasure such performance begets an actual misery.

On the other hand, let one of those of whom we said they could not be spoiled by the machinations of tutors, sit at a piano. But a few chords are needed to convince the assembled company that here is something not to be interrupted—something which must not be lost—something that conveys more joy than the most brilliant conversation—something that hushes the listener as though he were spoken to from some superior plane, and one not to be denied an attentive ear. At the conclusion of such music, there is a long drawn breath, not of relief that it is over, but of longing that there might be more to come.

The test of true music is in the soul, not in the head. We may profess to enjoy the one and affect contempt for the other, but the darkey with his banjo, and the backwoodsman with his fiddle will set people agog, and loosen up their ankle-joints far more readily, take the world as you find it, than any of your pianistic torturers or violinic prestidigitators.

It is to be regretted that art, the province of which is to delight the aesthetic faculty, should become a source of unhappiness to so many. We sometimes have doubts as to whether the kind of music to which we have alluded as the hyperartistic, really gives pleasure to any, even the musically fanatical. But it would be unjust to say that there are not lunatics in music as in everything else. There is no art or science, ology or ism under heaven, over which men have not gone crazy, and why not over music? Yet we claim it to be decidedly unjust to the sane, that the crazy ones should be allowed to convert the world into a grand musical lunatic asylum.

Honest, now, how many of those who profess to go into ecstasies over the performances of those gentlemen with the unpronounceable names who come over to us from Germany, and France, and Italy, can really tell, if blindfolded, and led into a concert hall, whether the pianist with the big name is fingering the keyboard, or four lively dogs are pawing the same in the vain endeavor to snatch as many shin-bones suspended over it. There are a few, whose veracity it would be very impolite—to say the least—to dispute, who claim that in these so-called grand productions, an occasional gleam of a something bearing some sort of a dim and undefined relation to a very vague sort of an air is sometimes caught running through the piece, and that they rather enjoy finding it, losing it, getting now and then glimpses of it, and finally giving it up like a conundrum. There are doubtless that kind of people in the world, and their enjoyment of this process is possibly quite keen, just as there are men who enjoy sitting around the stove of a country store, on old kegs or soap-boxes, and delight in playing "pick or po" with three pins apiece, and when one gets all, then divide and begin over again. There is no accounting for the peculiarities of mind we find everywhere about us. There are unquestionably those, too, who especially delight in seeing how very unhappy other people are made by anything of the annoying kind, and who are perfectly willing to undergo a large amount of torture themselves if they can experience the exquisite delight alluded to.

It is a fact that a very large majority of those who attend the "grand concerts" of to day, do so under protest. They do not enjoy them, but must go to sustain their reputation as cultivated patrons of music. They must anticipate the occasion with expressions of exquisite expectancy. They must deny themselves the relief of remaining at home, because their taste may be impugned. They prefer to endure the misery of a two hours submission to an ordeal of incomprehension, rather than the suspicion that they are not connoisseurs in music. Away from home and beyond the possibility of detection, they would choose to attend a minstrel performance rather than the most elaborate concert by the most distinguished artists of foreign importation. This is less a discredit to those who make this choice than to those who compel them to make it. If art has carried music beyond the range of the average comprehension, and society demands that its members must profess for that sort of thing an admiration which they cannot feel, the conspiracy between musical artists and the leaders of society is that against which we should inveigh. It makes hypocrites of us. It makes martyrs of us. It sometimes makes fools of us. What we need is an independence in matters of taste. We lean too much upon the opinions of wrongly acknowledged critics, who are often in league with the folly we ought to combat. It is no disgrace to differ from others in our tastes. Why then conceal that difference? We do not compromise our opinions half so much by candid expressions, as by formulating them after the model of anothers. If there be those who really enjoy the kind of music which is being presented upon the concert stage at this day—and it is possible there are such—let them sustain it if they can; but we protest against making those *particeps criminis* in the matter to whom such music is an abomination. The demand of the time is a return to something more simple—to melody—to harmony—to the comprehensible in music. We want sweetness rather than cleverness. We want the soul inspiring, instead of the brain racking. There is that which tickles the abnormally erudite in music, and evokes a sort of admiration for the process by which it is revealed; and there is that which unites with the latent love for music in every soul, and meets a joyous welcome for itself alone, regardless of the producing phenomena. It is more of the latter that we want.

In Pinafore, and kindred opera, we hail a dim foreshadowing of such a return. The great mass of people have placed their endorsement upon this class of music, which proves the direction in which the public sentiment is moving. In the contemptuous elevation of the hypercritical nose over Pinafore, we get a clew to the character of the condemners of a musical reform, but it only remains for us to encourage just such simple and beautiful compositions to achieve what this article urges.

